# Supplementary material for: Using lexical language models to detect borrowings in monolingual wordlists
Source: PLoS One. 2020 Dec 9;15(12):e0242709. doi: 10.1371/journal.pone.0242709 (PMC7725347; doi:10.1371/journal.pone.0242709)
Supplement: S1 Table — Borrowing rates of 5%, 10%, and 20%. (PDF) [file pone.0242709.s001.pdf]

**S1 Table. Detection results by language for seeded borrowings**

| | Recurrent neural net | | | | Markov model | | | | Bag of sounds | | | |
|---|---|---|---|---|---|---|---|---|---|---|---|---|
| **Language** | **Prec.** | **Recall** | **F1** | **Acc.** | **Prec.** | **Recall** | **F1** | **Acc.** | **Prec.** | **Recall** | **F1** | **Acc.** |
| Archi | 0.89 | 0.53 | 0.67 | 0.96 | 1.00 | 0.39 | 0.56 | 0.95 | 0.71 | 1.00 | 0.83 | 0.98 |
| Bezhta | 1.00 | 0.80 | 0.89 | 0.99 | 1.00 | 0.41 | 0.58 | 0.93 | 0.75 | 1.00 | 0.86 | 0.99 |
| Ceq Wong | 1.00 | 1.00 | 1.00 | 1.00 | 1.00 | 0.23 | 0.38 | 0.76 | 0.83 | 1.00 | 0.91 | 0.99 |
| Dutch | 0.71 | 0.71 | 0.71 | 0.97 | 0.94 | 0.60 | 0.73 | 0.96 | 0.67 | 1.00 | 0.80 | 0.98 |
| English | 0.86 | 0.75 | 0.80 | 0.98 | 0.89 | 0.24 | 0.37 | 0.86 | 0.80 | 1.00 | 0.89 | 0.99 |
| Gawwada | 0.93 | 0.82 | 0.87 | 0.98 | 1.00 | 0.54 | 0.70 | 0.95 | 0.73 | 1.00 | 0.84 | 0.99 |
| Gurindji | 1.00 | 1.00 | 1.00 | 1.00 | 1.00 | 1.00 | 1.00 | 1.00 | 0.93 | 1.00 | 0.96 | 0.99 |
| Hausa | 1.00 | 0.85 | 0.92 | 0.99 | 1.00 | 0.83 | 0.91 | 0.99 | 0.71 | 1.00 | 0.83 | 0.98 |
| Hawaiian | 1.00 | 1.00 | 1.00 | 1.00 | 1.00 | 1.00 | 1.00 | 1.00 | 1.00 | 1.00 | 1.00 | 1.00 |
| Hup | 0.88 | 1.00 | 0.93 | 1.00 | 1.00 | 0.31 | 0.48 | 0.95 | 0.86 | 1.00 | 0.92 | 1.00 |
| Imbabura Quechua | 1.00 | 1.00 | 1.00 | 1.00 | 0.89 | 0.89 | 0.89 | 0.99 | 0.89 | 1.00 | 0.94 | 0.99 |
| Indonesian | 1.00 | 0.95 | 0.97 | 1.00 | 0.92 | 0.92 | 0.92 | 0.99 | 0.92 | 1.00 | 0.96 | 1.00 |
| Iraqw | 1.00 | 0.83 | 0.91 | 0.99 | 0.93 | 0.81 | 0.87 | 0.98 | 0.80 | 1.00 | 0.89 | 0.99 |
| Japanese | 1.00 | 1.00 | 1.00 | 1.00 | 1.00 | 0.81 | 0.89 | 0.99 | 1.00 | 1.00 | 1.00 | 1.00 |
| Kali'na | 1.00 | 1.00 | 1.00 | 1.00 | 0.94 | 1.00 | 0.97 | 1.00 | 0.93 | 1.00 | 0.96 | 1.00 |
| Kanuri | 1.00 | 0.93 | 0.96 | 1.00 | 0.88 | 0.70 | 0.78 | 0.99 | 0.76 | 1.00 | 0.87 | 0.99 |
| Ket | 1.00 | 0.73 | 0.85 | 0.98 | 0.93 | 0.57 | 0.70 | 0.95 | 0.79 | 1.00 | 0.88 | 0.99 |
| Kildin Saami | 1.00 | 0.81 | 0.90 | 0.99 | 1.00 | 0.45 | 0.62 | 0.96 | 0.86 | 1.00 | 0.92 | 0.99 |
| Lower Sorbian | 1.00 | 0.87 | 0.93 | 0.99 | 0.92 | 0.57 | 0.71 | 0.97 | 0.71 | 1.00 | 0.83 | 0.98 |
| Malagasy | 1.00 | 1.00 | 1.00 | 1.00 | 1.00 | 0.88 | 0.93 | 0.99 | 1.00 | 1.00 | 1.00 | 1.00 |
| Manange | 1.00 | 0.79 | 0.88 | 0.99 | 0.88 | 0.41 | 0.56 | 0.90 | 0.82 | 1.00 | 0.90 | 0.99 |
| Mandarin Chinese | 1.00 | 0.94 | 0.97 | 1.00 | 1.00 | 0.91 | 0.95 | 1.00 | 0.74 | 1.00 | 0.85 | 0.99 |
| Mapudungun | 1.00 | 1.00 | 1.00 | 1.00 | 1.00 | 0.38 | 0.55 | 0.96 | 0.91 | 1.00 | 0.95 | 1.00 |
| Old High German | 0.82 | 0.60 | 0.69 | 0.97 | 1.00 | 0.54 | 0.70 | 0.96 | 0.81 | 1.00 | 0.90 | 0.99 |
| Oroqen | 1.00 | 0.47 | 0.64 | 0.96 | 1.00 | 0.48 | 0.65 | 0.94 | 0.41 | 1.00 | 0.58 | 0.96 |
| Otomi | 1.00 | 0.90 | 0.95 | 1.00 | 0.97 | 0.88 | 0.92 | 0.99 | 0.78 | 1.00 | 0.88 | 0.99 |
| Q'eqchi' | 0.94 | 0.70 | 0.80 | 0.98 | 0.94 | 0.62 | 0.74 | 0.97 | 0.71 | 1.00 | 0.83 | 0.99 |
| Romanian | 0.86 | 0.63 | 0.73 | 0.97 | 0.86 | 0.60 | 0.71 | 0.97 | 0.58 | 1.00 | 0.73 | 0.97 |
| Sakha | 1.00 | 0.83 | 0.91 | 0.98 | 1.00 | 0.68 | 0.81 | 0.98 | 0.71 | 1.00 | 0.83 | 0.98 |
| Saramaccan | 1.00 | 0.67 | 0.80 | 0.98 | 1.00 | 0.81 | 0.90 | 0.98 | 0.55 | 1.00 | 0.71 | 0.97 |
| Selice Romani | 1.00 | 1.00 | 1.00 | 1.00 | 0.89 | 0.33 | 0.48 | 0.89 | 0.71 | 1.00 | 0.83 | 0.99 |
| Seychelles Creole | 1.00 | 0.88 | 0.93 | 0.99 | 0.90 | 0.76 | 0.83 | 0.98 | 0.78 | 1.00 | 0.88 | 0.99 |
| Swahili | 1.00 | 0.93 | 0.97 | 1.00 | 1.00 | 0.90 | 0.95 | 0.99 | 0.91 | 1.00 | 0.95 | 1.00 |
| Takia | 1.00 | 0.80 | 0.89 | 0.99 | 0.82 | 0.47 | 0.60 | 0.94 | 0.91 | 1.00 | 0.95 | 1.00 |
| Tarifiyt Berber | 1.00 | 0.64 | 0.78 | 0.97 | 1.00 | 0.39 | 0.56 | 0.94 | 0.56 | 1.00 | 0.71 | 0.98 |
| Thai | 1.00 | 0.84 | 0.91 | 0.99 | 0.86 | 0.86 | 0.86 | 0.99 | 0.76 | 1.00 | 0.87 | 0.99 |
| Vietnamese | 1.00 | 0.63 | 0.77 | 0.97 | 1.00 | 0.50 | 0.67 | 0.97 | 0.77 | 1.00 | 0.87 | 0.99 |
| White Hmong | 1.00 | 0.85 | 0.92 | 0.99 | 1.00 | 1.00 | 1.00 | 1.00 | 0.95 | 1.00 | 0.97 | 1.00 |
| Wichí | 1.00 | 1.00 | 1.00 | 1.00 | 1.00 | 1.00 | 1.00 | 1.00 | 0.82 | 1.00 | 0.90 | 0.99 |
| Yaqui | 1.00 | 1.00 | 1.00 | 1.00 | 1.00 | 0.95 | 0.97 | 1.00 | 0.93 | 1.00 | 0.96 | 1.00 |
| Zinacantán Tzotzil | 1.00 | 0.92 | 0.96 | 1.00 | 0.91 | 0.77 | 0.83 | 0.98 | 0.91 | 1.00 | 0.95 | 1.00 |
| **Mean over languages** | 0.97 | 0.84 | 0.90 | 0.99 | 0.96 | 0.67 | 0.76 | 0.96 | 0.80 | 1.00 | 0.88 | 0.99 |

**Table 1.** Seeded borrowings - 5% borrowing - metrics by language.

| | Recurrent neural net | | | | Markov model | | | | Bag of sounds | | | |
|---|---|---|---|---|---|---|---|---|---|---|---|---|
| **Language** | **Prec** | **Recall** | **F1** | **Acc.** | **Prec.** | **Recall** | **F1** | **Acc.** | **Prec.** | **Recall** | **F1** | **Acc.** |
| Archi | 0.95 | 0.95 | 0.95 | 0.99 | 0.95 | 0.58 | 0.72 | 0.94 | 0.86 | 0.95 | 0.90 | 0.98 |
| Bezhta | 1.00 | 0.85 | 0.92 | 0.98 | 0.95 | 0.76 | 0.84 | 0.97 | 0.87 | 1.00 | 0.93 | 0.99 |
| Ceq Wong | 1.00 | 0.90 | 0.95 | 0.99 | 0.92 | 0.57 | 0.71 | 0.93 | 0.78 | 1.00 | 0.88 | 0.99 |
| Dutch | 0.82 | 0.78 | 0.79 | 0.94 | 0.86 | 0.71 | 0.77 | 0.95 | 0.72 | 0.95 | 0.82 | 0.97 |
| English | 1.00 | 0.83 | 0.91 | 0.98 | 0.88 | 0.70 | 0.78 | 0.94 | 0.75 | 1.00 | 0.86 | 0.99 |
| Gawwada | 0.96 | 0.92 | 0.94 | 0.99 | 0.96 | 0.74 | 0.83 | 0.96 | 0.86 | 1.00 | 0.92 | 0.99 |
| Gurindji | 1.00 | 0.96 | 0.98 | 0.99 | 1.00 | 1.00 | 1.00 | 1.00 | 0.89 | 1.00 | 0.94 | 0.99 |
| Hausa | 1.00 | 1.00 | 1.00 | 1.00 | 1.00 | 0.91 | 0.95 | 0.99 | 0.87 | 1.00 | 0.93 | 0.99 |
| Hawaiian | 1.00 | 1.00 | 1.00 | 1.00 | 1.00 | 1.00 | 1.00 | 1.00 | 0.97 | 1.00 | 0.98 | 1.00 |
| Hup | 1.00 | 0.92 | 0.96 | 0.99 | 0.96 | 0.85 | 0.90 | 0.98 | 0.84 | 1.00 | 0.91 | 0.99 |
| Imbabura Quechua | 1.00 | 1.00 | 1.00 | 1.00 | 1.00 | 1.00 | 1.00 | 1.00 | 0.96 | 1.00 | 0.98 | 1.00 |
| Indonesian | 1.00 | 1.00 | 1.00 | 1.00 | 0.95 | 0.95 | 0.95 | 0.99 | 0.88 | 1.00 | 0.94 | 0.99 |
| Iraqw | 1.00 | 0.96 | 0.98 | 1.00 | 0.96 | 0.79 | 0.87 | 0.97 | 0.91 | 1.00 | 0.95 | 0.99 |
| Japanese | 1.00 | 1.00 | 1.00 | 1.00 | 1.00 | 0.94 | 0.97 | 0.99 | 1.00 | 1.00 | 1.00 | 1.00 |
| Kali'na | 1.00 | 1.00 | 1.00 | 1.00 | 0.93 | 0.97 | 0.95 | 0.99 | 0.93 | 1.00 | 0.97 | 0.99 |
| Kanuri | 0.97 | 1.00 | 0.98 | 1.00 | 0.97 | 0.97 | 0.97 | 0.99 | 0.86 | 0.96 | 0.91 | 0.98 |
| Ket | 0.89 | 0.86 | 0.87 | 0.97 | 1.00 | 0.81 | 0.90 | 0.98 | 0.75 | 0.96 | 0.84 | 0.96 |
| Kildin Saami | 0.97 | 0.91 | 0.94 | 0.98 | 0.97 | 0.78 | 0.86 | 0.96 | 0.71 | 0.95 | 0.82 | 0.97 |
| Lower Sorbian | 0.93 | 0.96 | 0.95 | 0.99 | 1.00 | 0.97 | 0.99 | 1.00 | 0.88 | 1.00 | 0.94 | 0.99 |
| Malagasy | 1.00 | 1.00 | 1.00 | 1.00 | 1.00 | 0.94 | 0.97 | 0.99 | 0.85 | 1.00 | 0.92 | 0.98 |
| Manange | 0.96 | 1.00 | 0.98 | 1.00 | 1.00 | 0.82 | 0.90 | 0.98 | 0.96 | 1.00 | 0.98 | 1.00 |
| Mandarin Chinese | 0.98 | 0.98 | 0.98 | 1.00 | 1.00 | 0.98 | 0.99 | 1.00 | 0.89 | 1.00 | 0.94 | 0.99 |
| Mapudungun | 1.00 | 0.90 | 0.95 | 0.99 | 1.00 | 0.96 | 0.98 | 1.00 | 0.95 | 1.00 | 0.98 | 1.00 |
| Old High German | 0.85 | 0.71 | 0.77 | 0.96 | 0.81 | 0.84 | 0.82 | 0.97 | 0.86 | 0.95 | 0.90 | 0.98 |
| Oroqen | 0.89 | 0.86 | 0.87 | 0.97 | 1.00 | 0.70 | 0.82 | 0.96 | 0.55 | 1.00 | 0.71 | 0.96 |
| Otomi | 1.00 | 0.98 | 0.99 | 1.00 | 0.98 | 0.98 | 0.98 | 1.00 | 0.94 | 0.98 | 0.96 | 0.99 |
| Q'eqchi' | 0.96 | 0.90 | 0.92 | 0.98 | 0.97 | 0.94 | 0.95 | 0.99 | 0.95 | 1.00 | 0.97 | 0.99 |
| Romanian | 0.96 | 0.83 | 0.89 | 0.98 | 0.97 | 0.85 | 0.91 | 0.97 | 0.86 | 1.00 | 0.93 | 0.99 |
| Sakha | 0.90 | 0.96 | 0.93 | 0.98 | 0.92 | 0.83 | 0.87 | 0.97 | 0.80 | 1.00 | 0.89 | 0.99 |
| Saramaccan | 1.00 | 0.87 | 0.93 | 0.98 | 1.00 | 0.94 | 0.97 | 0.99 | 0.93 | 1.00 | 0.97 | 0.99 |
| Selice Romani | 0.94 | 0.88 | 0.91 | 0.98 | 0.84 | 0.94 | 0.89 | 0.98 | 0.80 | 1.00 | 0.89 | 0.98 |
| Seychelles Creole | 0.95 | 0.91 | 0.93 | 0.99 | 0.95 | 0.84 | 0.89 | 0.98 | 0.95 | 1.00 | 0.97 | 1.00 |
| Swahili | 0.97 | 1.00 | 0.99 | 1.00 | 1.00 | 0.97 | 0.98 | 1.00 | 0.92 | 0.96 | 0.94 | 0.99 |
| Takia | 1.00 | 0.80 | 0.89 | 0.98 | 0.93 | 0.93 | 0.93 | 0.98 | 0.95 | 1.00 | 0.97 | 1.00 |
| Tarifiyt Berber | 0.94 | 0.94 | 0.94 | 0.99 | 0.95 | 0.74 | 0.83 | 0.96 | 0.85 | 1.00 | 0.92 | 0.98 |
| Thai | 0.95 | 0.82 | 0.88 | 0.97 | 0.97 | 0.85 | 0.91 | 0.98 | 0.67 | 0.96 | 0.79 | 0.97 |
| Vietnamese | 0.97 | 1.00 | 0.99 | 1.00 | 1.00 | 0.97 | 0.98 | 1.00 | 0.90 | 1.00 | 0.95 | 0.99 |
| White Hmong | 1.00 | 1.00 | 1.00 | 1.00 | 1.00 | 0.96 | 0.98 | 1.00 | 0.92 | 1.00 | 0.96 | 0.99 |
| Wichí | 1.00 | 1.00 | 1.00 | 1.00 | 1.00 | 1.00 | 1.00 | 1.00 | 1.00 | 1.00 | 1.00 | 1.00 |
| Yaqui | 0.95 | 1.00 | 0.98 | 1.00 | 1.00 | 1.00 | 1.00 | 1.00 | 0.91 | 1.00 | 0.96 | 0.99 |
| Zinacantán Tzotzil | 1.00 | 0.97 | 0.98 | 1.00 | 0.90 | 0.78 | 0.84 | 0.97 | 0.80 | 1.00 | 0.89 | 0.98 |
| **Mean over languages** | 0.97 | 0.93 | 0.95 | 0.99 | 0.96 | 0.87 | 0.91 | 0.98 | 0.87 | 0.99 | 0.92 | 0.99 |

**Table 2.** Seeded borrowings - 10% borrowing - metrics by language.

| | Recurrent neural net | | | | Markov model | | | | Bag of sounds | | | |
|---|---|---|---|---|---|---|---|---|---|---|---|---|
| **Language** | **Prec.** | **Recall** | **F1** | **Acc.** | **Prec.** | **Recall** | **F1** | **Acc.** | **Prec.** | **Recall** | **F1** | **Acc.** |
| Archi | 0.98 | 0.98 | 0.98 | 0.99 | 0.96 | 0.94 | 0.95 | 0.98 | 0.88 | 0.93 | 0.91 | 0.96 |
| Bezhta | 0.98 | 0.97 | 0.97 | 0.99 | 0.96 | 0.96 | 0.96 | 0.98 | 0.96 | 0.96 | 0.96 | 0.98 |
| Ceq Wong | 0.97 | 0.85 | 0.91 | 0.96 | 0.94 | 0.91 | 0.92 | 0.97 | 0.89 | 1.00 | 0.94 | 0.98 |
| Dutch | 0.88 | 0.77 | 0.82 | 0.92 | 0.83 | 0.78 | 0.81 | 0.91 | 0.78 | 1.00 | 0.88 | 0.96 |
| English | 0.93 | 0.89 | 0.91 | 0.97 | 1.00 | 0.80 | 0.89 | 0.96 | 0.83 | 1.00 | 0.91 | 0.97 |
| Gawwada | 0.97 | 1.00 | 0.98 | 0.99 | 0.95 | 0.93 | 0.94 | 0.98 | 0.93 | 1.00 | 0.96 | 0.98 |
| Gurindji | 1.00 | 0.98 | 0.99 | 1.00 | 1.00 | 0.97 | 0.99 | 1.00 | 0.92 | 1.00 | 0.96 | 0.98 |
| Hausa | 0.98 | 1.00 | 0.99 | 1.00 | 0.98 | 0.98 | 0.98 | 0.99 | 0.93 | 0.99 | 0.96 | 0.98 |
| Hawaiian | 1.00 | 1.00 | 1.00 | 1.00 | 0.98 | 1.00 | 0.99 | 1.00 | 0.99 | 1.00 | 0.99 | 1.00 |
| Hup | 1.00 | 1.00 | 1.00 | 1.00 | 0.98 | 0.98 | 0.98 | 0.99 | 0.82 | 1.00 | 0.90 | 0.97 |
| Imbabura Quechua | 1.00 | 1.00 | 1.00 | 1.00 | 0.97 | 1.00 | 0.99 | 1.00 | 0.93 | 1.00 | 0.96 | 0.99 |
| Indonesian | 1.00 | 1.00 | 1.00 | 1.00 | 0.97 | 0.97 | 0.97 | 0.99 | 0.97 | 1.00 | 0.99 | 0.99 |
| Iraqw | 0.96 | 0.96 | 0.96 | 0.99 | 0.96 | 0.96 | 0.96 | 0.99 | 0.88 | 1.00 | 0.94 | 0.98 |
| Japanese | 1.00 | 1.00 | 1.00 | 1.00 | 0.99 | 0.97 | 0.98 | 0.99 | 0.94 | 1.00 | 0.97 | 0.99 |
| Kali'na | 1.00 | 1.00 | 1.00 | 1.00 | 0.98 | 1.00 | 0.99 | 1.00 | 0.96 | 1.00 | 0.98 | 0.99 |
| Kanuri | 0.98 | 0.98 | 0.98 | 0.99 | 0.98 | 1.00 | 0.99 | 1.00 | 0.90 | 1.00 | 0.95 | 0.98 |
| Ket | 1.00 | 1.00 | 1.00 | 1.00 | 0.95 | 0.90 | 0.92 | 0.97 | 0.93 | 0.98 | 0.95 | 0.98 |
| Kildin Saami | 1.00 | 0.96 | 0.98 | 0.99 | 0.91 | 0.84 | 0.87 | 0.95 | 0.92 | 0.98 | 0.95 | 0.98 |
| Lower Sorbian | 1.00 | 0.99 | 0.99 | 1.00 | 0.98 | 0.96 | 0.97 | 0.99 | 0.92 | 1.00 | 0.96 | 0.98 |
| Malagasy | 1.00 | 0.99 | 0.99 | 1.00 | 1.00 | 1.00 | 1.00 | 1.00 | 0.97 | 1.00 | 0.98 | 0.99 |
| Manange | 1.00 | 0.98 | 0.99 | 1.00 | 1.00 | 0.89 | 0.94 | 0.98 | 0.92 | 1.00 | 0.96 | 0.98 |
| Mandarin Chinese | 1.00 | 0.97 | 0.98 | 0.99 | 1.00 | 0.98 | 0.99 | 1.00 | 0.93 | 1.00 | 0.96 | 0.98 |
| Mapudungun | 1.00 | 0.98 | 0.99 | 1.00 | 1.00 | 1.00 | 1.00 | 1.00 | 0.96 | 0.98 | 0.97 | 0.99 |
| Old High German | 0.96 | 0.82 | 0.89 | 0.96 | 0.91 | 0.81 | 0.86 | 0.95 | 0.89 | 0.94 | 0.92 | 0.97 |
| Oroqen | 1.00 | 0.98 | 0.99 | 1.00 | 0.86 | 0.85 | 0.85 | 0.94 | 0.67 | 0.92 | 0.78 | 0.93 |
| Otomi | 0.99 | 0.97 | 0.98 | 0.99 | 1.00 | 0.99 | 1.00 | 1.00 | 0.91 | 0.98 | 0.95 | 0.98 |
| Q'eqchi' | 1.00 | 0.97 | 0.98 | 0.99 | 0.99 | 0.95 | 0.97 | 0.99 | 0.90 | 1.00 | 0.95 | 0.98 |
| Romanian | 0.94 | 0.93 | 0.93 | 0.97 | 0.90 | 0.90 | 0.90 | 0.96 | 0.85 | 0.92 | 0.88 | 0.96 |
| Sakha | 0.98 | 0.97 | 0.98 | 0.99 | 0.96 | 0.94 | 0.95 | 0.98 | 0.81 | 1.00 | 0.89 | 0.96 |
| Saramaccan | 1.00 | 0.94 | 0.97 | 0.99 | 1.00 | 0.96 | 0.98 | 0.99 | 0.90 | 0.93 | 0.92 | 0.98 |
| Selice Romani | 1.00 | 0.95 | 0.97 | 0.99 | 1.00 | 0.87 | 0.93 | 0.97 | 0.91 | 1.00 | 0.95 | 0.98 |
| Seychelles Creole | 0.99 | 0.99 | 0.99 | 1.00 | 0.96 | 0.93 | 0.95 | 0.98 | 0.89 | 1.00 | 0.94 | 0.98 |
| Swahili | 1.00 | 0.99 | 0.99 | 1.00 | 1.00 | 1.00 | 1.00 | 1.00 | 0.89 | 0.98 | 0.94 | 0.98 |
| Takia | 0.96 | 0.96 | 0.96 | 0.98 | 1.00 | 0.89 | 0.94 | 0.98 | 0.91 | 1.00 | 0.95 | 0.98 |
| Tarifiyt Berber | 1.00 | 0.98 | 0.99 | 1.00 | 0.91 | 0.89 | 0.90 | 0.96 | 0.94 | 0.92 | 0.93 | 0.98 |
| Thai | 0.98 | 0.94 | 0.96 | 0.98 | 0.96 | 0.88 | 0.92 | 0.97 | 0.88 | 1.00 | 0.94 | 0.98 |
| Vietnamese | 1.00 | 1.00 | 1.00 | 1.00 | 1.00 | 0.99 | 0.99 | 1.00 | 0.96 | 1.00 | 0.98 | 0.99 |
| White Hmong | 1.00 | 1.00 | 1.00 | 1.00 | 1.00 | 1.00 | 1.00 | 1.00 | 1.00 | 1.00 | 1.00 | 1.00 |
| Wichí | 1.00 | 0.96 | 0.98 | 0.99 | 1.00 | 0.98 | 0.99 | 1.00 | 1.00 | 1.00 | 1.00 | 1.00 |
| Yaqui | 0.99 | 1.00 | 0.99 | 1.00 | 0.98 | 0.98 | 0.98 | 0.99 | 0.94 | 1.00 | 0.97 | 0.99 |
| Zinacantán Tzotzil | 1.00 | 0.98 | 0.99 | 1.00 | 0.98 | 1.00 | 0.99 | 1.00 | 0.91 | 1.00 | 0.96 | 0.98 |
| **Mean over languages** | 0.99 | 0.97 | 0.98 | 0.99 | 0.97 | 0.94 | 0.95 | 0.98 | 0.91 | 0.99 | 0.94 | 0.98 |

**Table 3.** Seeded borrowings - 20% borrowing - metrics by language.
