# Supplementary material for: Using lexical language models to detect borrowings in monolingual wordlists
Source: PLoS One. 2020 Dec 9;15(12):e0242709. doi: 10.1371/journal.pone.0242709 (PMC7725347; doi:10.1371/journal.pone.0242709)
Supplement: S2 Table — Cross-validation means and standard deviations. (PDF) [file pone.0242709.s002.pdf]

## S2 Table. Ten-fold cross validation of detection results by language for WOLD wordlists

| | Recurrent neural net | | | | Markov model | | | | Bag of Sounds | | | | |
|---|---|---|---|---|---|---|---|---|---|---|---|---|---|
| **Language** | **Prec.** | **Recall** | **F1** | **Acc** | **Prec.** | **Recall** | **F1** | **Acc** | **Prec.** | **Recall** | **F1** | **Acc** | **Native** |
| Archi | 0.747 | 0.603 | 0.664 | 0.840 | 0.740 | 0.530 | 0.615 | 0.803 | 0.249 | 0.687 | 0.359 | 0.814 | 0.786 |
| Bezhta | 0.799 | 0.759 | 0.778 | 0.863 | 0.770 | 0.704 | 0.735 | 0.834 | 0.689 | 0.757 | 0.720 | 0.840 | 0.701 |
| Ceq Wong | 0.770 | 0.710 | 0.736 | 0.818 | 0.690 | 0.619 | 0.650 | 0.753 | 0.581 | 0.649 | 0.610 | 0.754 | 0.667 |
| Dutch | 0.625 | 0.492 | 0.545 | 0.807 | 0.557 | 0.432 | 0.484 | 0.778 | 0.010 | 0.200 | 0.019 | 0.815 | 0.814 |
| English | 0.651 | 0.624 | 0.637 | 0.716 | 0.630 | 0.601 | 0.613 | 0.696 | 0.510 | 0.685 | 0.584 | 0.720 | 0.614 |
| Gawwada | 0.661 | 0.367 | 0.457 | 0.864 | 0.590 | 0.357 | 0.438 | 0.862 | 0.179 | 0.603 | 0.254 | 0.916 | 0.909 |
| Gurindji | 0.428 | 0.232 | 0.291 | 0.753 | 0.525 | 0.301 | 0.376 | 0.796 | 0.000 | 0.000 | 0.000 | 0.875 | 0.875 |
| Hausa | 0.654 | 0.527 | 0.579 | 0.813 | 0.702 | 0.520 | 0.595 | 0.814 | 0.288 | 0.664 | 0.395 | 0.830 | 0.802 |
| Hawaiian | 0.783 | 0.430 | 0.554 | 0.797 | 0.727 | 0.475 | 0.572 | 0.826 | 0.022 | 0.400 | 0.041 | 0.843 | 0.839 |
| Hup | 0.869 | 0.629 | 0.727 | 0.936 | 0.807 | 0.527 | 0.625 | 0.903 | 0.464 | 0.908 | 0.593 | 0.939 | 0.899 |
| Imbabura Quechua | 0.861 | 0.780 | 0.816 | 0.892 | 0.859 | 0.791 | 0.821 | 0.897 | 0.615 | 0.762 | 0.676 | 0.839 | 0.720 |
| Indonesian | 0.689 | 0.619 | 0.651 | 0.778 | 0.657 | 0.608 | 0.630 | 0.768 | 0.265 | 0.636 | 0.371 | 0.732 | 0.698 |
| Iraqw | 0.787 | 0.560 | 0.647 | 0.894 | 0.690 | 0.459 | 0.545 | 0.855 | 0.377 | 0.760 | 0.493 | 0.901 | 0.870 |
| Japanese | 0.822 | 0.723 | 0.767 | 0.853 | 0.767 | 0.703 | 0.733 | 0.835 | 0.413 | 0.680 | 0.513 | 0.768 | 0.704 |
| Kali'na | 0.714 | 0.506 | 0.590 | 0.857 | 0.660 | 0.537 | 0.589 | 0.868 | 0.208 | 0.967 | 0.335 | 0.885 | 0.855 |
| Kanuri | 0.635 | 0.444 | 0.518 | 0.794 | 0.598 | 0.429 | 0.493 | 0.788 | 0.055 | 0.683 | 0.101 | 0.830 | 0.823 |
| Ket | 0.779 | 0.470 | 0.582 | 0.906 | 0.683 | 0.402 | 0.486 | 0.885 | 0.207 | 0.750 | 0.315 | 0.930 | 0.916 |
| Kildin Saami | 0.560 | 0.454 | 0.497 | 0.790 | 0.562 | 0.403 | 0.469 | 0.758 | 0.003 | 0.050 | 0.006 | 0.805 | 0.810 |
| Lower Sorbian | 0.744 | 0.605 | 0.664 | 0.856 | 0.713 | 0.602 | 0.649 | 0.849 | 0.215 | 0.752 | 0.328 | 0.831 | 0.803 |
| Malagasy | 0.602 | 0.373 | 0.452 | 0.821 | 0.559 | 0.365 | 0.437 | 0.821 | 0.000 | 0.000 | 0.000 | 0.875 | 0.875 |
| Manange | 0.593 | 0.293 | 0.380 | 0.881 | 0.638 | 0.274 | 0.360 | 0.859 | 0.045 | 0.300 | 0.079 | 0.937 | 0.935 |
| Mandarin Chinese | 0.050 | 0.006 | 0.011 | 0.955 | 0.190 | 0.008 | 0.016 | 0.811 | 0.000 | 0.000 | 0.000 | 0.993 | 0.993 |
| Mapudungun | 0.816 | 0.664 | 0.727 | 0.879 | 0.801 | 0.636 | 0.707 | 0.868 | 0.534 | 0.832 | 0.645 | 0.885 | 0.800 |
| Old High German | 0.351 | 0.189 | 0.241 | 0.887 | 0.450 | 0.176 | 0.246 | 0.860 | 0.000 | 0.000 | 0.000 | 0.947 | 0.947 |
| Oroqen | 0.530 | 0.271 | 0.354 | 0.857 | 0.497 | 0.267 | 0.342 | 0.856 | 0.054 | 0.350 | 0.091 | 0.925 | 0.922 |
| Otomi | 0.908 | 0.709 | 0.793 | 0.954 | 0.929 | 0.629 | 0.749 | 0.939 | 0.673 | 0.854 | 0.751 | 0.957 | 0.902 |
| Q'eqchi' | 0.852 | 0.651 | 0.733 | 0.935 | 0.820 | 0.597 | 0.689 | 0.923 | 0.540 | 0.816 | 0.647 | 0.939 | 0.895 |
| Romanian | 0.724 | 0.698 | 0.710 | 0.764 | 0.716 | 0.668 | 0.690 | 0.743 | 0.412 | 0.623 | 0.493 | 0.663 | 0.600 |
| Sakha | 0.632 | 0.599 | 0.610 | 0.800 | 0.620 | 0.543 | 0.577 | 0.782 | 0.196 | 0.595 | 0.290 | 0.766 | 0.751 |
| Saramaccan | 0.622 | 0.589 | 0.603 | 0.714 | 0.632 | 0.596 | 0.611 | 0.718 | 0.089 | 0.669 | 0.149 | 0.652 | 0.645 |
| Selice Romani | 0.872 | 0.905 | 0.888 | 0.874 | 0.875 | 0.878 | 0.876 | 0.859 | 0.829 | 0.746 | 0.784 | 0.740 | 0.427 |
| Seychelles Creole | 0.568 | 0.272 | 0.364 | 0.828 | 0.606 | 0.323 | 0.420 | 0.854 | 0.000 | 0.000 | 0.000 | 0.911 | 0.911 |
| Swahili | 0.783 | 0.680 | 0.723 | 0.857 | 0.700 | 0.623 | 0.658 | 0.825 | 0.536 | 0.786 | 0.635 | 0.851 | 0.758 |
| Takia | 0.808 | 0.618 | 0.697 | 0.839 | 0.762 | 0.617 | 0.680 | 0.834 | 0.047 | 0.700 | 0.087 | 0.780 | 0.768 |
| Tarifiyt Berber | 0.764 | 0.795 | 0.778 | 0.788 | 0.765 | 0.772 | 0.768 | 0.774 | 0.695 | 0.773 | 0.731 | 0.750 | 0.511 |
| Thai | 0.655 | 0.526 | 0.581 | 0.799 | 0.622 | 0.450 | 0.521 | 0.754 | 0.104 | 0.630 | 0.175 | 0.794 | 0.785 |
| Vietnamese | 0.668 | 0.463 | 0.544 | 0.795 | 0.579 | 0.411 | 0.477 | 0.770 | 0.101 | 0.601 | 0.166 | 0.821 | 0.817 |
| White Hmong | 0.597 | 0.373 | 0.457 | 0.785 | 0.607 | 0.354 | 0.443 | 0.767 | 0.025 | 0.400 | 0.046 | 0.846 | 0.845 |
| Wichí | 0.873 | 0.705 | 0.773 | 0.931 | 0.848 | 0.729 | 0.781 | 0.935 | 0.518 | 0.698 | 0.583 | 0.900 | 0.857 |
| Yaqui | 0.819 | 0.736 | 0.773 | 0.885 | 0.839 | 0.764 | 0.798 | 0.897 | 0.567 | 0.794 | 0.658 | 0.861 | 0.760 |
| Zinacantán Tzotzil | 0.906 | 0.751 | 0.815 | 0.940 | 0.836 | 0.675 | 0.744 | 0.919 | 0.430 | 0.935 | 0.584 | 0.913 | 0.857 |
| **Mean over languages** | 0.697 | 0.546 | 0.603 | 0.844 | 0.678 | 0.521 | 0.578 | 0.828 | 0.286 | 0.578 | 0.349 | 0.843 | 0.797 |

**Table 1.** 10-fold cross-validation by language - Means.

| | Recurrent neural net | | | | Markov model | | | | Bag of Sounds | | | |
|---|---|---|---|---|---|---|---|---|---|---|---|---|
| **Language** | **Prec.** | **Recall** | **F1** | **Acc.** | **Prec.** | **Recall** | **F1** | **Acc.** | **Prec.** | **Recall** | **F1** | **Acc.** |
| Archi | 0.073 | 0.071 | 0.052 | 0.029 | 0.107 | 0.093 | 0.087 | 0.045 | 0.078 | 0.099 | 0.084 | 0.031 |
| Bezhta | 0.052 | 0.035 | 0.035 | 0.030 | 0.037 | 0.054 | 0.040 | 0.029 | 0.045 | 0.070 | 0.044 | 0.033 |
| Ceq Wong | 0.044 | 0.104 | 0.072 | 0.047 | 0.115 | 0.089 | 0.089 | 0.066 | 0.083 | 0.085 | 0.072 | 0.053 |
| Dutch | 0.149 | 0.105 | 0.103 | 0.051 | 0.076 | 0.069 | 0.061 | 0.042 | 0.016 | 0.350 | 0.031 | 0.030 |
| English | 0.046 | 0.051 | 0.042 | 0.021 | 0.062 | 0.073 | 0.055 | 0.043 | 0.029 | 0.047 | 0.024 | 0.018 |
| Gawwada | 0.177 | 0.093 | 0.080 | 0.042 | 0.101 | 0.107 | 0.100 | 0.029 | 0.158 | 0.379 | 0.201 | 0.021 |
| Gurindji | 0.170 | 0.094 | 0.099 | 0.046 | 0.234 | 0.126 | 0.151 | 0.031 | 0.000 | 0.000 | 0.000 | 0.036 |
| Hausa | 0.081 | 0.066 | 0.048 | 0.028 | 0.079 | 0.095 | 0.086 | 0.033 | 0.098 | 0.138 | 0.109 | 0.036 |
| Hawaiian | 0.064 | 0.054 | 0.057 | 0.032 | 0.086 | 0.077 | 0.078 | 0.037 | 0.031 | 0.516 | 0.057 | 0.030 |
| Hup | 0.122 | 0.086 | 0.089 | 0.019 | 0.138 | 0.158 | 0.120 | 0.035 | 0.170 | 0.102 | 0.151 | 0.024 |
| Imbabura Quechua | 0.065 | 0.046 | 0.035 | 0.022 | 0.053 | 0.062 | 0.037 | 0.018 | 0.107 | 0.092 | 0.084 | 0.037 |
| Indonesian | 0.035 | 0.061 | 0.045 | 0.029 | 0.058 | 0.059 | 0.047 | 0.034 | 0.067 | 0.085 | 0.078 | 0.041 |
| Iraqw | 0.155 | 0.146 | 0.134 | 0.031 | 0.134 | 0.091 | 0.095 | 0.025 | 0.093 | 0.126 | 0.080 | 0.022 |
| Japanese | 0.041 | 0.060 | 0.034 | 0.022 | 0.045 | 0.017 | 0.021 | 0.022 | 0.068 | 0.092 | 0.076 | 0.047 |
| Kali'na | 0.095 | 0.087 | 0.084 | 0.029 | 0.087 | 0.122 | 0.104 | 0.035 | 0.084 | 0.105 | 0.118 | 0.027 |
| Kanuri | 0.089 | 0.071 | 0.060 | 0.041 | 0.126 | 0.077 | 0.085 | 0.038 | 0.039 | 0.328 | 0.068 | 0.024 |
| Ket | 0.107 | 0.099 | 0.100 | 0.023 | 0.150 | 0.178 | 0.148 | 0.033 | 0.132 | 0.362 | 0.180 | 0.023 |
| Kildin Saami | 0.093 | 0.099 | 0.087 | 0.024 | 0.042 | 0.038 | 0.036 | 0.043 | 0.010 | 0.158 | 0.019 | 0.028 |
| Lower Sorbian | 0.089 | 0.084 | 0.072 | 0.031 | 0.103 | 0.059 | 0.062 | 0.035 | 0.084 | 0.110 | 0.106 | 0.041 |
| Malagasy | 0.137 | 0.095 | 0.083 | 0.035 | 0.087 | 0.097 | 0.089 | 0.035 | 0.000 | 0.000 | 0.000 | 0.020 |
| Manange | 0.162 | 0.126 | 0.137 | 0.026 | 0.176 | 0.140 | 0.143 | 0.038 | 0.073 | 0.483 | 0.127 | 0.025 |
| Mandarin Chinese | 0.158 | 0.019 | 0.033 | 0.019 | 0.341 | 0.014 | 0.026 | 0.041 | 0.000 | 0.000 | 0.000 | 0.007 |
| Mapudungun | 0.105 | 0.105 | 0.087 | 0.040 | 0.092 | 0.103 | 0.089 | 0.037 | 0.092 | 0.124 | 0.085 | 0.027 |
| Old High German | 0.156 | 0.060 | 0.078 | 0.023 | 0.147 | 0.066 | 0.081 | 0.024 | 0.000 | 0.000 | 0.000 | 0.025 |
| Oroqen | 0.137 | 0.091 | 0.101 | 0.035 | 0.173 | 0.103 | 0.117 | 0.031 | 0.078 | 0.474 | 0.130 | 0.021 |
| Otomi | 0.074 | 0.112 | 0.091 | 0.019 | 0.057 | 0.059 | 0.048 | 0.016 | 0.090 | 0.054 | 0.069 | 0.011 |
| Q'eqchi' | 0.076 | 0.101 | 0.075 | 0.023 | 0.073 | 0.094 | 0.084 | 0.020 | 0.106 | 0.114 | 0.104 | 0.018 |
| Romanian | 0.028 | 0.037 | 0.022 | 0.019 | 0.037 | 0.045 | 0.031 | 0.030 | 0.056 | 0.060 | 0.038 | 0.030 |
| Sakha | 0.073 | 0.074 | 0.047 | 0.029 | 0.112 | 0.116 | 0.110 | 0.035 | 0.069 | 0.154 | 0.088 | 0.027 |
| Saramaccan | 0.074 | 0.093 | 0.080 | 0.037 | 0.069 | 0.058 | 0.053 | 0.044 | 0.048 | 0.224 | 0.068 | 0.044 |
| Selice Romani | 0.022 | 0.029 | 0.017 | 0.019 | 0.021 | 0.031 | 0.018 | 0.019 | 0.042 | 0.033 | 0.026 | 0.025 |
| Seychelles Creole | 0.089 | 0.048 | 0.046 | 0.025 | 0.114 | 0.048 | 0.064 | 0.016 | 0.000 | 0.000 | 0.000 | 0.031 |
| Swahili | 0.072 | 0.089 | 0.057 | 0.020 | 0.074 | 0.054 | 0.057 | 0.027 | 0.061 | 0.070 | 0.051 | 0.028 |
| Takia | 0.059 | 0.070 | 0.044 | 0.019 | 0.081 | 0.090 | 0.084 | 0.056 | 0.046 | 0.483 | 0.082 | 0.033 |
| Tarifiyt Berber | 0.054 | 0.042 | 0.033 | 0.029 | 0.039 | 0.045 | 0.031 | 0.033 | 0.053 | 0.062 | 0.045 | 0.042 |
| Thai | 0.055 | 0.052 | 0.038 | 0.018 | 0.069 | 0.045 | 0.045 | 0.030 | 0.054 | 0.091 | 0.077 | 0.019 |
| Vietnamese | 0.073 | 0.064 | 0.053 | 0.030 | 0.095 | 0.119 | 0.110 | 0.035 | 0.061 | 0.262 | 0.095 | 0.035 |
| White Hmong | 0.139 | 0.073 | 0.091 | 0.031 | 0.150 | 0.101 | 0.110 | 0.038 | 0.043 | 0.516 | 0.076 | 0.038 |
| Wichí | 0.103 | 0.123 | 0.090 | 0.024 | 0.048 | 0.089 | 0.060 | 0.016 | 0.150 | 0.129 | 0.129 | 0.024 |
| Yaqui | 0.067 | 0.069 | 0.054 | 0.031 | 0.046 | 0.066 | 0.035 | 0.024 | 0.072 | 0.069 | 0.053 | 0.018 |
| Zinacantán Tzotzil | 0.060 | 0.097 | 0.041 | 0.021 | 0.060 | 0.098 | 0.077 | 0.025 | 0.102 | 0.093 | 0.102 | 0.028 |
| **Pooled std. dev.** | 0.100 | 0.082 | 0.072 | 0.030 | 0.114 | 0.088 | 0.082 | 0.034 | 0.078 | 0.226 | 0.088 | 0.030 |

**Table 2.** 10-fold cross-validation by language - Standard Deviations.
